# Successful treatment of mycobacterial infection associated hemophagocytic lymphohistiocytosis with etoposide and anti-tuberculous therapy: a case report

**DOI:** 10.1186/s12879-020-05016-4

**Published:** 2020-05-05

**Authors:** Yan-Hong Wang, Jun-Hui Ba, Xiao-Wei Shi, Ben-Quan Wu

**Affiliations:** Department of Medical Intensive Care Unit, the Third Affiliated Hospital of Sun Yat-Sen University, No. 600 Tian He Road, Guangzhou, 510630 Guangdong Province China

**Keywords:** Hemophagocytic lymphohistiocytosis, *Mycobacterium* infection, Tuberculosis, Etoposide

## Abstract

**Background:**

Hemophagocytic lymphohistiocytosis (HLH) is a rare and potentially life-threatening disorder characterized by an exacerbated but ineffective inflammatory response, which can be classified as primary and secondary HLH. HLH associated with *Mycobacterium tuberculosis* is uncommon. This case report accounted an immunocompetent patient who was confirmed to be *Mycobacterium* infection, or rather, highly suspected tuberculosis (TB) associated HLH, with a favorable outcome.

**Case presentation:**

A 36-year-old man presented with persistent fever, pancytopenia, and hyperferritinemia. A bone marrow smear demonstrated hemophagocytosis, and pathological examination of lung biopsy was positive for acid-fast bacilli, which established the diagnosis of *Mycobacterium* infection and HLH. Then the patient treated successfully with anti-TB therapy, along with 8 weeks of etoposide.

**Conclusion:**

This case emphasizes that HLH should be kept in mind when clinicians encounter a patient with severe infection presenting with pancytopenia and hyperferritinemia. Given the high mortality, early diagnosis and appropriate therapy can provide patients with a favorable prognosis.

## Background

Hemophagocytic lymphohistiocytosis (HLH) is a fatal hyper-inflammatory syndrome induced by aggressive activation of macrophages and cytotoxic T cells and natural killer (NK) cells, resulting in hypercytokinemia and immune-mediated injury of multiple organ systems [1]. Clinical and laboratory features include fever, splenomegaly, neurological dysfunction, coagulopathy, liver dysfunction, cytopenia, hypertriglyceridemia, hyperferritinemia, hemophagocytosis, and diminished NK cell activity [2]. HLH is often classified as primary or as secondary. Primary HLH, accompanied by genetic mutations and poor prognosis, is most common in children. Secondary HLH is usually triggered by medical conditions, including infections, malignancies, autoimmune diseases, and immunodeficient states. Among infectious diseases, secondary HLH is commonly induced by viral infections, such as Epstein–Barr virus (EBV) infection. Occasionally, it is associated with bacterial, fungal, or parasitic infections. HLH is a rare and severe complication of tuberculosis (TB), with approximately 3% morbidity and 50% mortality [3]. Normally, previous TB-HLH cases were treated with anti-TB therapy. In some situation, the patients need more aggressive management. Herein, we present a patient who was confirmed to be *Mycobacterium* infection, or rather, highly suspected TB induced HLH, with a favorable outcome following early administration of anti-TB therapy combined with etoposide.

## Case presentation

**Fig. 1:**
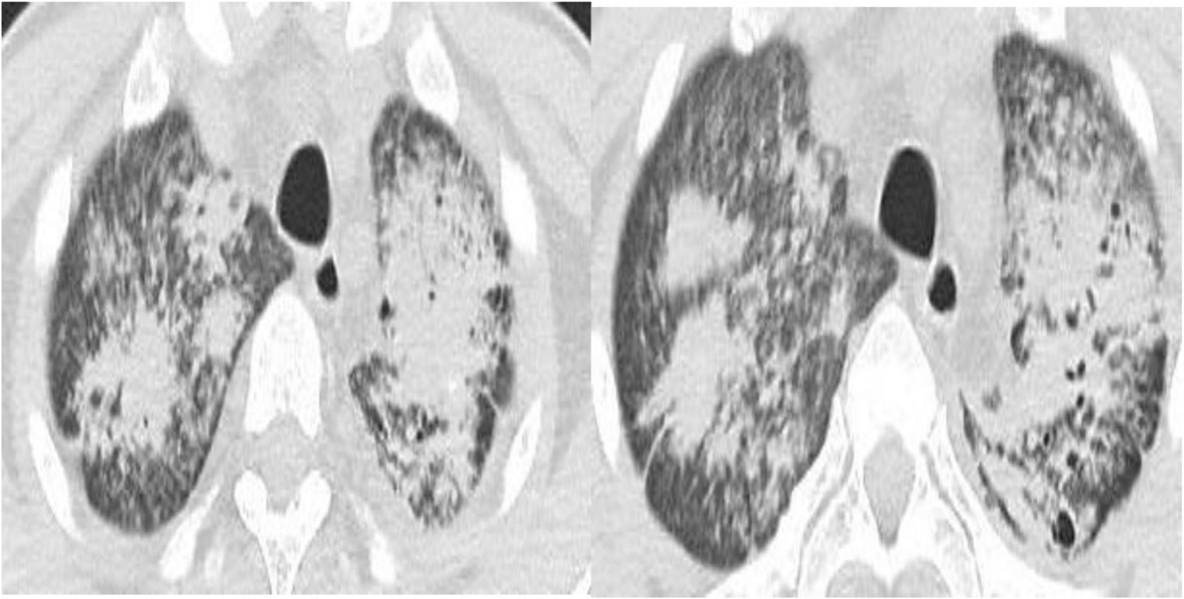
Chest CT illustrating diffuse infiltrates in both lungs; possible cavitation formed in the left upper lobe, with mediastinal and hilar lymph node enlargement

**Fig. 2:**
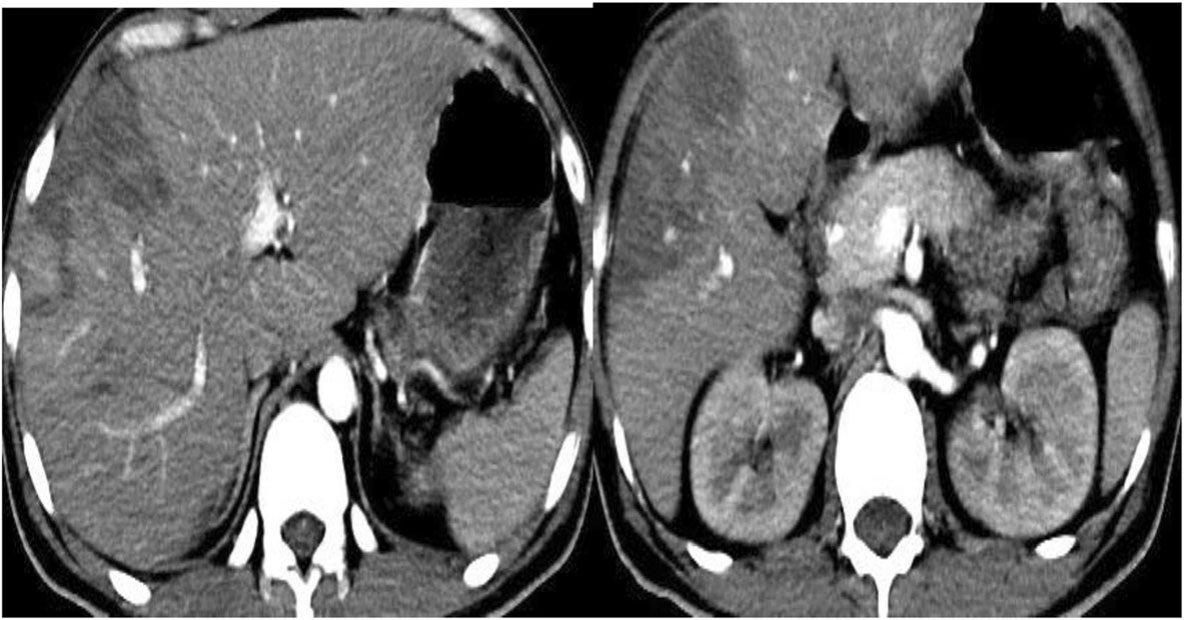
Abdominal CT showing multiple focal lesions in the liver

A previously healthy 36-year-old Chinese man presented to the emergency department with a 1-month history of fever. He had consulted a nearby doctor before arriving at our hospital. Symptomatic treatment was given but did not work. Chest computed tomography (CT) showed diffused bilateral infiltration (Fig. 1). Abdominal CT showed multiple focal lesions in the liver (Fig. 2), without hepatosplenomegaly. Because of rapidly clinical deterioration with onset of acute respiratory failure, he was transferred to the medical intensive care unit.

On admission, his vital signs were: temperature, 37.2 °C; heart rate, 119 beats/min; blood pressure (BP), 90/48 mmHg; and respiration rate, 38 breaths/min with oxygen saturation of 88%. The patient appeared to be acutely ill. A pulmonary examination revealed rales in both lungs. His abdomen was soft, with no hepatomegaly and splenomegaly. Routine laboratory tests results were: white blood cell (WBC) count, 5.32 × 10^9^ cells/L; hemoglobin, 94 g/L; platelet count, 70 × 10^9^/L; aspartate aminotransferase (AST), 121 U/L; alanine aminotransferase (ALT), 47 U/L; total bilirubin (TB), 48.4 μmol/L; direct bilirubin (DB), 40.9 μmol/L; blood urea nitrogen (BUN), 11.35 mmol/L; creatinine, 106.0 μmol/L; lactic dehydrogenase (LDH) 508 U/L; serum ferritin, 17,208.98 ng/ml. Even with continuous high-flow oxygen inhalation, arterial blood gas analysis showed pH 7.45, PO_2_ 57 mmHg, PCO_2_ 35 mmHg and lactate 2.0 mmol/L. Coagulation tests demonstrated prothrombin time (PT) 16.9 s, prothrombin activity (PTA) 62%, and fibrinogen level 1.74 g/L. Autoimmune antibody blood tests were negative. Microbiological and serological work-up for HIV, hepatitis A, B, C and E viruses, EBV, cytomegalovirus, dengue virus, malaria, *Leptospira*, and scrub typhus was negative. Bacteriological assays as well as serology for respiratory viruses (adenovirus, and influenza A and B, parainfluenza and respiratory syncytial viruses), *Mycoplasma pneumoniae*, *Chlamydia pneumoniae* and *Legionella* were also negative. Repeated sputum smear samples were negative for acid-fast bacilli. The patient had no significant past medical history, specifically no prior TB infections, chronic illnesses, liver or kidney disease, transfusions, malignancy, or immune diseases. He was not taking any regular prescription and he had not travelled abroad.

After admission, the patient was initially diagnosed with fever of unknown origin, pulmonary infection. According to chest and abdominal CT findings, pulmonary TB and hepatic abscess were highly suspected. Malignancy was excluded by positron emission tomography. Considering possible multisite infections due to hypervirulent *Klebsiella pneumoniae*, we started an antibiotic regimen with meropenem and amikacin. Non-invasive positive pressure ventilation was applied for respiratory support. However, the patient continued to present with recurrent high-grade fever. Peripheral blood TB ELISPOT assay was positive, which suggested TB. Anti-TB therapy with rifapentine (0.6 g once a week), isoniazid (0.3 g daily), ethambutol (0.75 g daily) and moxifloxacin (0.4 g daily) was administered.

**Fig. 3:**
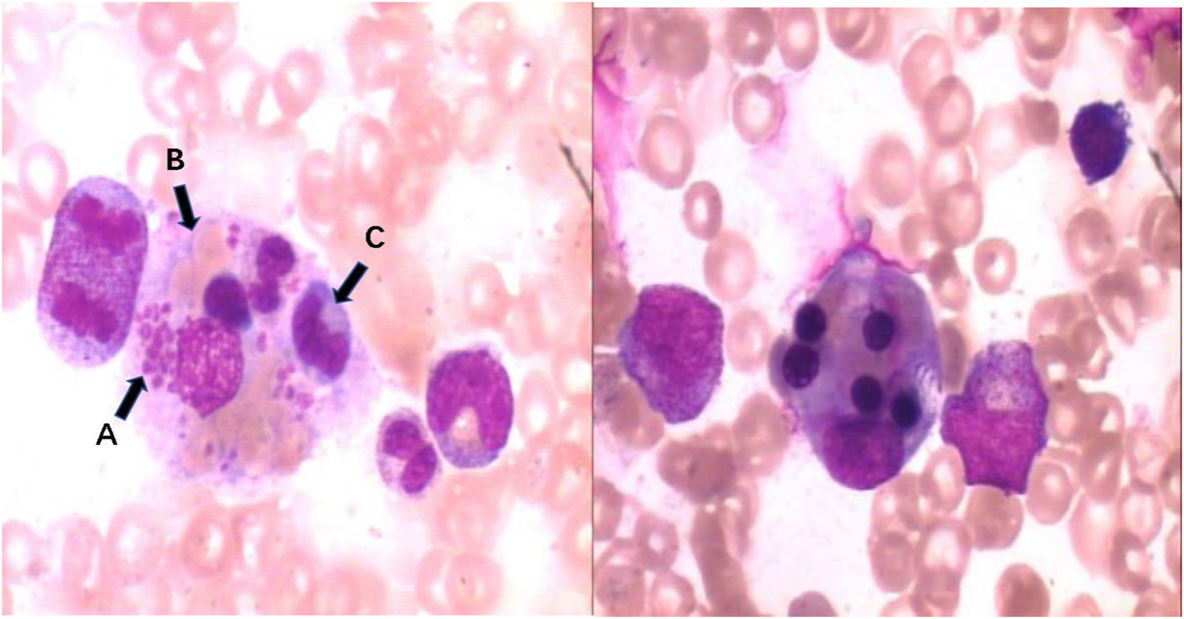
Bone marrow smear showing platelets (A), red cells (B) and neutrophil (C) engulfed by macrophages

The patient’s clinical course was notable for obvious dyspnea, intermittent fever, worsening pancytopenia, and hyperferritinemia during observation (Table 1). On day 4, examination of bone marrow aspiration was undertaken, which revealed increased macrophage activity with hemophagocytosis (Fig. 3). Additional blood tests showed low NK cell activity (11.9%, normal value: ≥15.1%) and high-soluble CD25 levels (> 44,000 pg/ml, normal value: < 2400 pg/ml). Accordingly, a diagnosis of HLH was made. However, the patient refused to undergo genetic testing. Following consultation with a hematologist, intravenous etoposide was started on day 7 (0.1 g twice a week for 2 weeks, 0.1 g once a week for the following 6 weeks). The patient responded well to the therapeutic strategy, with marked improvement of fever and cytopenia. On day 17, he was successfully withdrawn from the ventilator.

**Table 1:** Laboratory examination results during the clinical course

Parameter	Day 1	Day 5	Day 9	Day 13	Day 17	Day 53	Day 64	Normal value
**White blood cell count (10**^**9**^**/L)**	1.25	1.8	1.95	1.62	4.19	86.47	6.29	3.5–9.5
**Hemoglobin (g/L)**	78	72	75	91	80	94	83	130–175
**Platelet count (10**^**9**^**/L)**	41	15	64	41	105	289	177	100–350
**Fibrinogen (g/L)**	1.79	1.85	3.39	4.94	–	–	3.77	2.0–4.0
**Ferritin (ng/mL)**	375,554	19,336	–	–	18,570	3361	2855	22–322

**Fig. 4:**
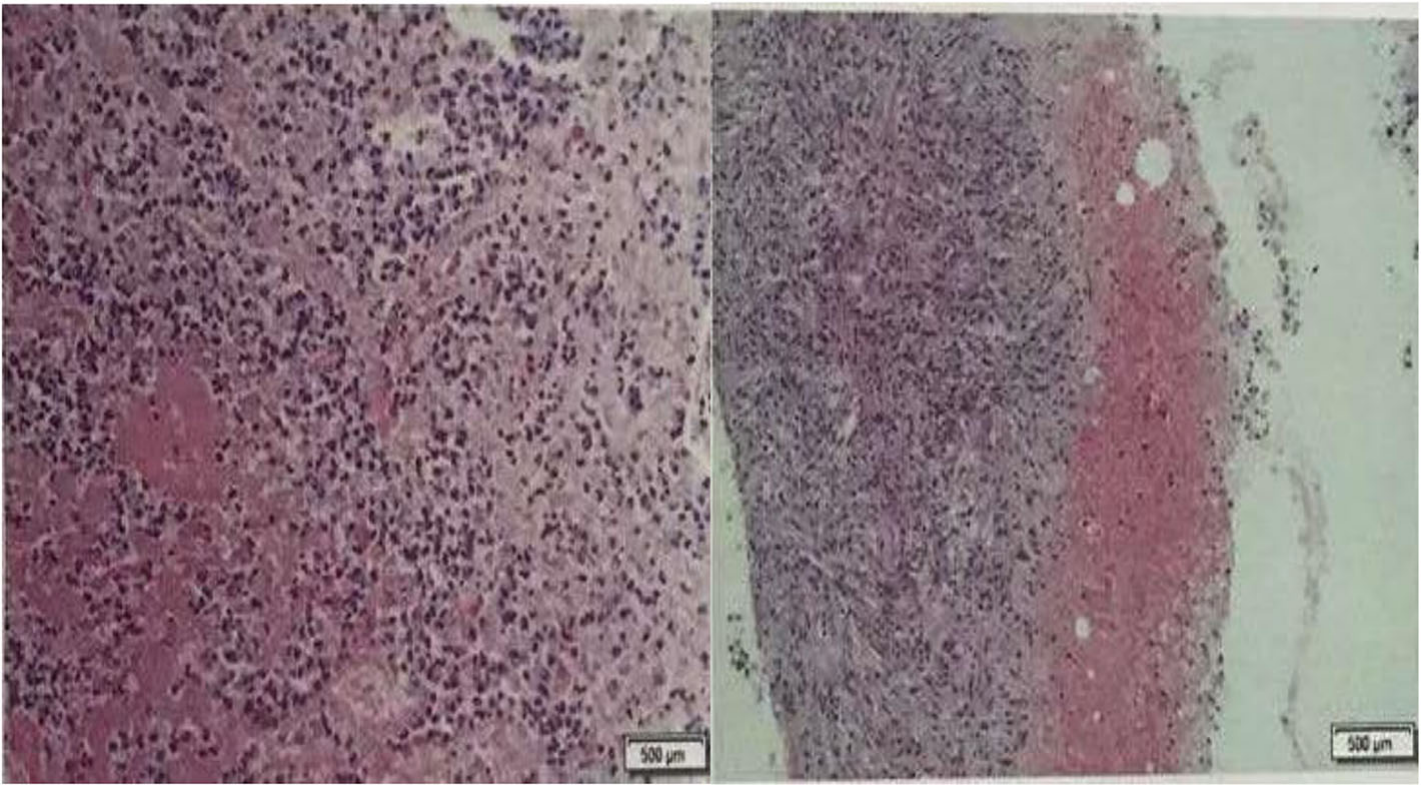
Pathological result of lung biopsy showing positive acid-fast bacilli

**Fig. 5:**
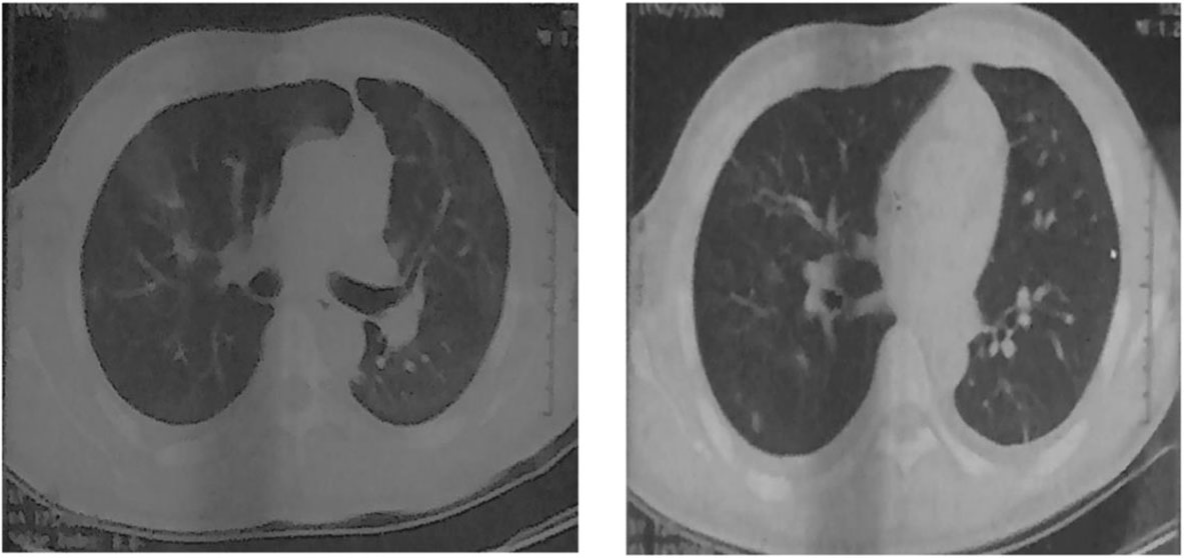
Repeated chest CT revealing infiltration markedly improved and fibrous proliferation, calcification

Once the patient was stable and free of symptoms of respiratory failure, he was transferred to the respiratory department on day 19. Anti-TB therapy with isoniazid, rifapentine, ethambutol and pyrazinamide was administered. After platelet recovery, ultrasound-guided percutaneous lung biopsy was performed on day 22. The pathological result was positive for acid-fast bacilli (Fig. 4), which suggested differentiating between *Mycobacterium tuberculosis* (MTB) and non-tuberculosis mycobacteria (NTM). Re-examination of bone marrow aspiration showed no hemophagocytosis. After discharge in good condition on day 67, he was followed up for 3 months in our hospital and treated with a course of four standard anti-TB drugs. The patient has stayed systemically well. Six months later, the repeated chest CT findings in another tertiary hospital revealed fibrous proliferation, calcification and pleural thickening, which still indicated tuberculosis (Fig. 5).

## Discussion and conclusion

Although viruses are the most frequent trigger in infection-associated HLH, *Mycobacterium tuberculosis* can act as an obligate intracellular pathogen. The underlying mechanism of TB-associated HLH remains to be elucidated. In China, TB is a serious public health issue, with the second highest incidence rate in the world. However, it is not easy to establish the diagnosis due to its diverse clinical manifestations, low sensitivity for acid-fast staining, and time-consuming culture. Polymerase chain reaction of peripheral blood or sputum has become an attractive diagnostic tool because of its high sensitivity. However, it is unavailable in our hospital. According to the patient’s radiological features and positive TB ELISPOT results, empirical anti-TB therapy was instituted. Given the low platelet counts, invasive procedures were not carried out at this time. After improvement of cytopenia, lung biopsy was performed, and the final pathological result suggested possible MTB or NTM infections. In a review of the literature of cases of HLH, there were no reports of HLH associated with NTM infection. Since lacking of enough etiology evidences focused on TB, the diagnosis of *Mycobacterium* infection was established. Nevertheless, we proposed that HLH was most likely to be secondary to TB in our patient. He underwent a course of rigorous anti-TB therapeutic regimen. Chest infiltration markedly improved and fibrous proliferation, calcification was observed in repeated CT features. According to medical image diagnostic report in another tertiary hospital, TB infection was still highly considered. It was generally known that the first line anti-TB drugs without combination of other antibiotics, such as clarithromycin or amikacin, was not sufficient for treatment of NTM infection.

HLH is known as a rare hematological syndrome with acute onset and poor prognosis with clinical deterioration of the condition. It is treated with immunosuppressants, etoposide, and allogeneic hematopoietic stem cell transplantation. EBV-HLH carries nearly 100% mortality in untreated cases. In contrast, in the patients with TB-HLH who do not receive any treatment or delayed treatment, the mortality is approximately 50%. However, most information on the treatment of HLH comes from the pediatric literature. No prospective trials guiding the first-line treatment of HLH in adults have taken place. Treatment regimens targeting hyper-inflammation are therefore based on pediatric protocols, such as HLH-94 and HLH-2004, which may result in over-treatment and unnecessary toxicity in adults [4]. With regard to TB-HLH, it normally depends on successfully treating TB alone. There is no doubt that rapid anti-TB therapy is the cornerstone of treatment and is crucial for preventing HLH in TB patients. In some situations, patients require immunomodulatory therapy, including steroids and intravenous immunoglobulin (IVIG). Most previous TB-HLH cases were treated with anti-TB therapy with or without steroids [5]. Our patient experienced rapid deterioration and required more intensive management. Anti-TB therapy alone seemed to be ineffective, since he had recurrent dyspnea, high-grade fever and cytopenia in the course of treatment. Moreover, we considered that HLH may have been another driver, together with TB, of the patient’s worsening condition. In such circumstances, prompt introduction of etoposide was recommended after consultation with an expert experienced in the treatment of HLH. In addition, dexamethasone was not used in this patient, since it is supposed to cause aggravation of potential infections.

In fact, etoposide treatment in TB-HLH remains controversial. Mbizvo et al. reported a TB-HLH patient who was successfully treated with 8 weeks of etoposide and dexamethasone. In contrast, Aggarwal reported a patient who died 2 weeks after use of steroid, etoposide and cyclosporine [6]. Etoposide, a classical chemotherapeutic agent in HLH-94 and HLH-04 treatment protocol, is generally believed to specifically inhibit the activation of the monocyte–macrophage system by ablating overactivation of T cells and suppressing production of inflammatory cytokines [7]. This differs from the wide immunosuppressive effects of corticosteroids and immunoregulation of IVIG. Initiation of etoposide is effective in severe EBV-infected or HIV-infected patients with HLH, but is less well known in other forms of infection-associated HLH. Meanwhile, it is usually not indicated in HLH induced by TB [4].

By reviewing this case, we propose that the decision to conduct HLH treatment depends on individualized clinical judgement and assessment of organ function. Appropriate timing of intervention, suitable doses, course of treatment, and toxicity require more consideration and clinical observation. It was unlikely that our patient would have improved his condition without early recognition of HLH and initiation of etoposide combined with anti-TB therapy.

In conclusion, we report a case of highly suspected TB infection induced HLH, successfully rescued by a combination therapy of anti-TB treatment and immunosuppression with etoposide. Given the high mortality, early and specific therapy targeting the underlying etiology is crucial in secondary HLH. Although it is not recommended in TB-HLH, etoposide can be attempted in refractory cases that fail to respond well to anti-TB treatment alone.

## Data Availability

The datasets used during the current study are available from the corresponding or last author on reasonable request.

## Abbreviations

HLH: Hemophagocytic lymphohistiocytosis
TB: Tuberculosis
NK: Natural killer
EBV: Epstein–Barr virus
CT: Computed tomography
BP: Blood pressure
WBC: White blood cell
AST: Aspartate aminotransferase
ALT: Alanine aminotransferase
TB: Total bilirubin
DB: Direct bilirubin
BUN: Blood urea nitrogen
LDH: Lactic dehydrogenase
PT: Prothrombin time
PTA: Prothrombin activity
IVIG: Intravenous immunoglobulin
MTB: *Mycobacterium tuberculosis*
NTM: Non-tuberculosis mycobacteria
